# Design and Performance Evaluation of a Rotary Magnetorheological Damper for Unmanned Vehicle Suspension Systems

**DOI:** 10.1155/2013/894016

**Published:** 2013-03-06

**Authors:** Jae-Hoon Lee, Changwan Han, Dongsu Ahn, Jin Kyoo Lee, Sang-Hu Park, Seonghun Park

**Affiliations:** ^1^School of Mechanical Engineering, Pusan National University, Jangjeon-dong, Geumjeong-gu, Busan 609-732, Republic of Korea; ^2^Suspension Systems Engineering Team, Doosan Corporation Mottrol, 456-3 Nae-dong, Seongsan-gu, Changwon, Gyeongnam 642-050, Republic of Korea

## Abstract

We designed and validated a rotary magnetorheological (MR) damper with a specified damping torque capacity, an unsaturated magnetic flux density (MFD), and a high magnetic field intensity (MFI) for unmanned vehicle suspension systems. In this study, for the rotary type MR damper to have these satisfactory performances, the roles of the sealing location and the cover case curvature of the MR damper were investigated by using the detailed 3D finite element model to reflect asymmetrical shapes and sealing components. The current study also optimized the damper cover case curvature based on the MFD, the MFI, and the weight of the MR damper components. The damping torques, which were computed using the characteristic equation of the MR fluid and the MFI of the MR damper, were 239.2, 436.95, and 576.78 N*·*m at currents of 0.5, 1, and 1.5 A, respectively, at a disk rotating speed of 10 RPM. These predicted damping torques satisfied the specified damping torque of 475 N*·*m at 1.5 A and showed errors of less than 5% when compared to experimental measurements from the MR damper manufactured by the proposed design. The current study could play an important role in improving the performance of rotary type MR dampers.

## 1. Introduction

Unmanned vehicles for combat can run and petrol automatically to defend against enemy attack and intrusion. Unmanned vehicles can drive at a speed of 50 km/h, although on the off-road, and have approximately a weight of 1 ton with various machine guns and sensors to attack and detect enemies as well as to navigate a path [1, 2]. Therefore, it is crucial that the vehicle body and equipped sensors of unmanned vehicles are under stable operating conditions by using robust suspension systems.

A damper is a major component of a vehicle suspension system that provides comfort and steering controllability of the vehicle [3]. Although electrically controlled suspension systems have been used to improve the dynamic performance of vehicles, such systems are still limited due to discontinuous damping forces, structural complexity, and high cost. To address these limitations, semiactive suspension systems with electronic-rheological (ER) and magneto-rheological (MR) fluids have been widely studied [4–7]. An MR fluid has variable yield shear stresses that depend on magnetic field intensity [8]. This characteristic is used to develop the design and control algorithms for various application systems [9–12]. Moreover, weight reduction and improvements in the performance of mechanical systems that use MR fluids have been studied recently [13–17].

The facts that a rotary MR damper (a rotary damper with an MR fluid) has a simple structure with a fast response speed driven by electronic control circuits [13, 18, 19] and that its low friction and wear increase the running life and stability of other mechanical systems as well as its own can satisfy the damper requirements for unmanned vehicle suspension systems [20–22]. Many previous studies have evaluated the MR damper performance by calculating the magnetic field intensity (MFI) through finite element (FE) analysis [13, 16, 18, 21, 23–30]. However, these studies have focused on reciprocating type MR dampers, not rotary types. Even previous studies regarding rotary type MR dampers have only focused on designing the overall shapes and rectangular cross-section dimensions of MR dampers, using simple 2D or 3D geometric models with a symmetric shape between the upper and lower sides for a rotating axis [10, 13, 21, 29–31].

Unlike related studies in the literature, the present study attempted to investigate the effect of sealing locations and cover case curvatures on the performance of a rotary type MR damper, thus optimizing these design parameters. Moreover, the asymmetric nature of the MR damper used in the study requires that a detailed 3D FE model needs to be generated and that the damping torque needs to be calculated individually from the upper and lower surfaces between the disk and MR fluid. Therefore, the first objective of this study is to investigate the sealing location and the optimized cover case curvature of a rotary type MR damper which could maximize the magnetic field intensity (MFI) as well as to minimize the weight of the MR damper with the magnetic flux density (MFD) lower than the saturation limit. The second objective is to determine if the damping torque capacity of the proposed MR damper design would be suitable for unmanned vehicle suspension systems and further to validate it through comparisons of the analytical predictions and experimental measurements of the damping torque capacity for the proposed optimal design.

## 2. Design Procedure

**Figure 1:**
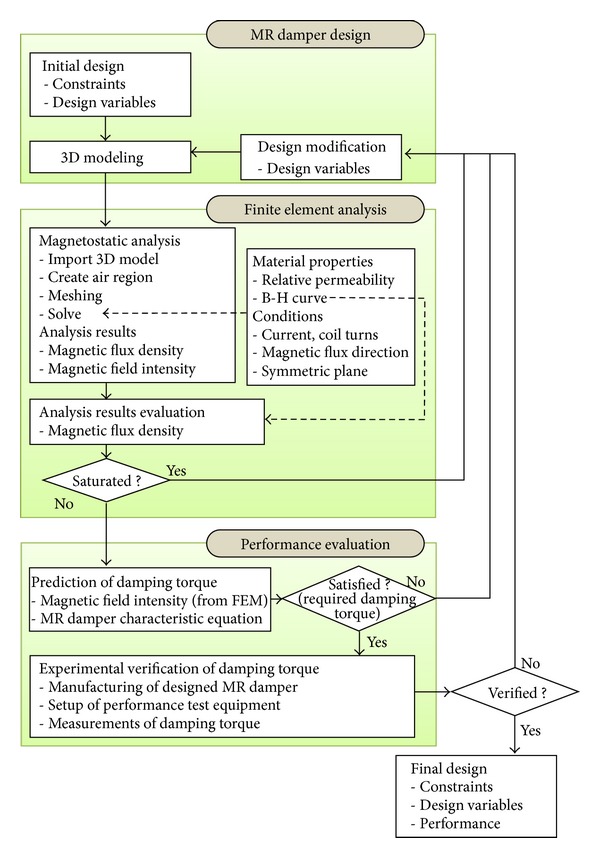
Design procedure of the rotary MR damper.

The main issues in the design of a rotary MR damper are to reduce the size and weight of its components to allow installation within a limited space and to determine the optimal dimensions of the components such that the MR fluid has the maximum magnetic field strength. Reducing the size and weight of a rotary MR damper decreases the required driving energy of an MR damper-installed system with a corresponding increase in the efficiency of the system. In addition, optimization of the component dimensions contributes to maximizing the damping torque capacity of an MR damper. The proposed rotary MR damper is required to produce a damping torque of more than 475 N·m using 400 coil turns and an input current of 1.5 A at a rotating speed of 10 RPM. Our detailed design procedure is shown in Figure 1.

Using ANSYS commercial finite element (FE) analysis software (V11, ANSYS Inc., USA), we constructed a three dimensional (3D) magnetostatic finite element model based on an initial design of the rotary MR damper [13, 18, 27]. The MFD and MFI values of the damper components were evaluated and compared to the saturation ranges of operation for the component materials. Changes in the design and MFD evaluations using magnetostatic FE analysis were repeated until the MFD values of all the components were shown to be unsaturated.

The damping torque of the proposed rotary MR damper was computed using the characteristic equation of the MR fluid and the MFI of the rotary MR damper. When the MR damper could not achieve the required damping torque capacity, further changes in the design were made. For the final design of the rotary MR damper, the damping torque was experimentally measured to validate the design procedure and the accuracy of our analytical methods.

## 3. Characteristic Equation of the MR Damper

Damping torque is generated by changes in friction between the rotating disk and MR fluid due to changes in the magnetically dependent viscosity of the MR fluid [8]. The magnetic field of the MR fluid is controlled electronically by the magnitude of the current (which depends on the number of solenoid coil turns) and structurally by the shape and dimensions of the ferromagnetic material around the coil.

**Figure 2:**
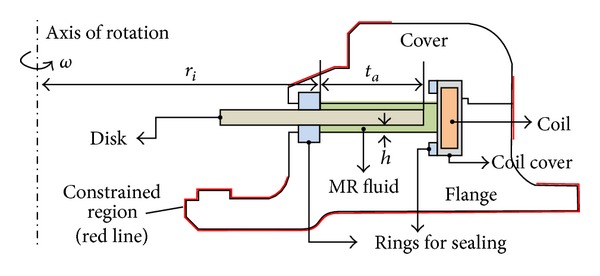
Cross-section of the rotary MR damper.

To achieve the desired damping torque capacity, the rotary MR damper is composed of single or multiple rotating disks connected to a rotating shaft, coils that generate a magnetic field, a sealed case to prevent the MR fluid from leaking outside by enclosing the coils, and various sealing rings (Figure 2). The case is divided into two parts, a flange and a cover, and can be detached and reattached. Both the case and the rotating disk have design limitations with respect to the external shape, depending on the details of their installation in a system. The design limitations are identified by the red line shown in Figure 2.

The total damping torque (*T*_total_) of the rotary MR damper is given by

(1) $$\begin{array}{c} T_{\text{total}}=T_{\text{vis}}+T_{\text{yd}}, \end{array}$$

where *T*_vis_ is the torque due to the viscosity of the MR fluid itself without applied current and *T*_yd_ is the torque due to increased viscosity with the increasing magnetic field strength of the MR fluid by the applied current.

MRF-1400CG (LORD Corporation, USA) was used as the MR fluid with viscosity (*η*) of 0.250 Pa·s [32]. *T*_vis_ is proportional to the rotational speed (*ω*) and the cross-sectional area of the MR fluid and is inversely proportional to the gap (*h*) between the rotational disk and the cover. *T*_vis_ can be approximated by [26]

(2) $$\begin{array}{c} T_{\text{vis}}=\pi \eta \frac{\omega }{2h}\left\{{\left(r_{i}+t_{a}\right)}^{4}-{\left(r_{i}\right)}^{4}\right\}, \end{array}$$

where *r*_*i*_ is the radius between the axis of rotation and the MR fluid and *t*_*a*_ is the radial distance of the MR fluid from the center axis exposed to the magnetic field.

*T*_yd_ is calculated as follows [9]:

(3) $$\begin{array}{c} T_{\text{yd}}=\frac{2\pi }{3}\tau \left(H\right)\left\{{\left(r_{i}+t_{a}\right)}^{3}-{\left(r_{i}\right)}^{3}\right\}, \end{array}$$

where *τ*(*H*) is the yield shear stress of the MR fluid and is a function of the MFI. *τ*(*H*) is given by the following experimentally derived equation from Carlson [9] and is proportional to the MFI of the gap between the rotational disk and the cover (*H*_MR_):

(4) $$\begin{array}{c} \tau \left(H\right)=C\times 271,700\times \Phi^{1.5239}\times \tanh\left(6.33\times 10^{-6}H_{\text{MR}}\right), \end{array}$$

where Φ is the iron particle fraction (0.4) (which is the volume ratio of iron particles to the MR fluid) and *C* is a coefficient dependent on the carrier fluid of the MR fluid (*C* = 1 in this study because the carrier fluid of MRF-140CG is hydrocarbon).

To design an efficient rotary MR damper with a high damping torque capacity, high MFI and unsaturated MFD values of the damper components and fluid are required.

## 4. Damping Torque Evaluation by Finite Element Analysis

### 4.1. Magnetostatic Finite Element Analysis

**Figure 3:**
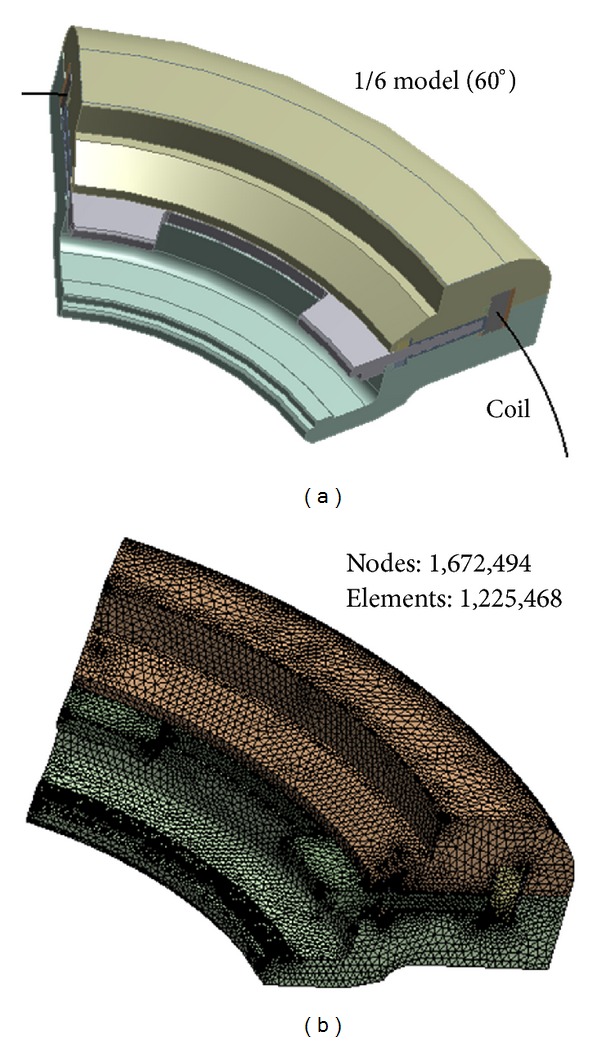
3D model of the rotary MR damper for FE analysis. (a) One-sixth MR damper model. (b) Finite element (FE) model.

A 3D FE model of the rotary MR damper was constructed using a one-sixth segment of the structure by considering axial symmetry, as shown in Figure 3(a). Most previous studies used 2D FE models to reduce the time and cost of generating and modifying geometric models as well as performing FE analysis. In this study, however, a 3D model with details of sealing components was generated due to the asymmetric nature of the damper for a vertical rotating axis, because those 2D models of previous studies can be only used when changes in the geometry are ignored in the circumferential direction [13, 33]. The coil turns used to generate electric current were modeled as line elements, and other components were modeled as solid elements. In addition, an external air region surrounding the entire MR damper was generated. The final FE model was constructed using tetrahedral meshes with approximately 120 million second-order elements and 160 million nodes, as shown in Figure 3(b).

**Figure 4:**
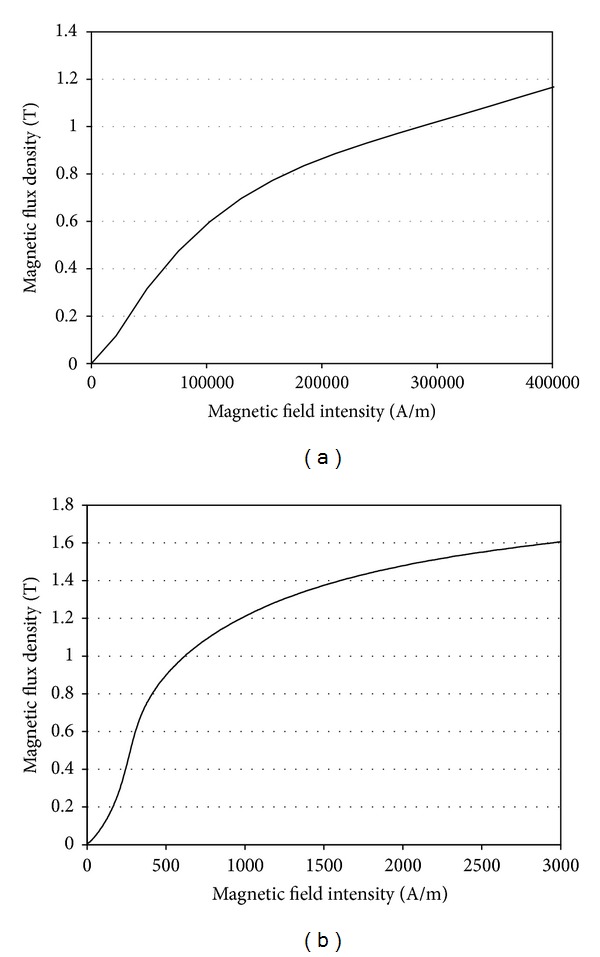
B-H curves of MR fluid and ferromagnetic materials. (a) MR Fluid (MRF-140CG). (b) Ferromagnetic material (SS-400).

The rotary MR damper components can be categorized into two material groups according to their magnetization properties. The rotational disk, flange, and cover are usually made of structural steel that can be easily magnetized by electric current and can be classified as ferromagnetic. Other components of the MR damper are not magnetizable and can be classified as paramagnetic. The relative permeability of paramagnetic materials, such as rubber for the sealing rings and copper for the coils, is close to 1, while that of the ferromagnetic materials is not a constant value and varies nonlinearly with the MFI. The nonlinear relative permeability values of the MR fluid (MRF-140CG) and cover case (SS-400) materials can be calculated from the slopes of the B-H curves (the MFD versus MFI curves) published in the literature (Figure 4) [32]. The relative permeability of ferromagnetic materials is much higher than paramagnetic materials, which contributes to a reduction in the volume of the rotary MR damper while allowing increased magnetic field strength. The relative permeability values of other paramagnetic components, including the surrounding air, are defined as 1.

**Figure 5:**
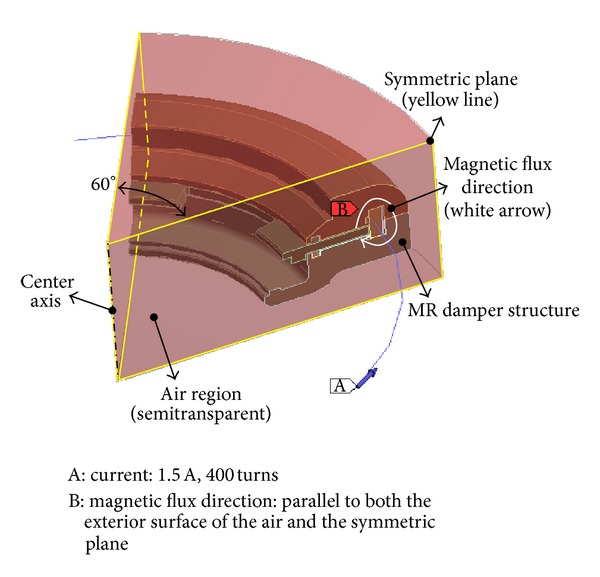
Load and boundary conditions.

In the magnetostatic FE analysis, 400 coil turns and an electric current of 1.5 A were modeled in the coil region (Figure 5). The magnetic field that formed in concentric circles around the coil where electric currents were applied was assumed to be parallel to the two symmetric planes (the edges cut diametrically to create a one-sixth model), since the coil direction is perpendicular to the symmetric planes. The direction of the magnetic field was also assumed to be parallel to the external surface of the surrounding air because most of the magnetic field is produced in the ferromagnetic material, not in the air (which is modeled as paramagnetic material). In addition, the air is located far from the coil. The boundary condition that the magnetic field is parallel to the FE model's surface is necessary for the magnetostatic FE analysis to be performed because it prevents the magnetic field from leaking outside the model's surface.

**Figure 6:**
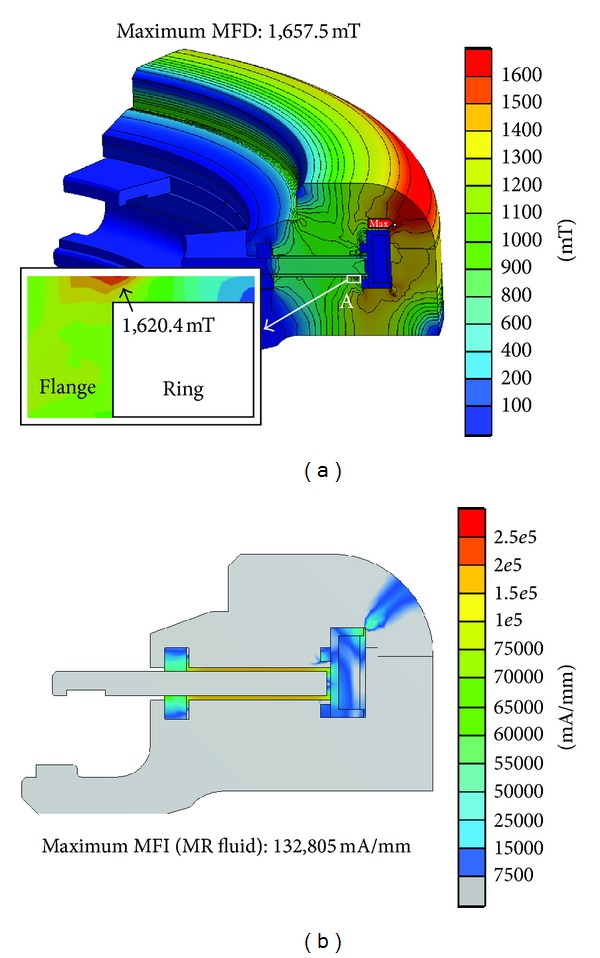
FEA results of the rotary MR damper (Model A). (a) Magnetic flux density (MFD). (b) Magnetic field intensity (MFI).

**Figure 7:**
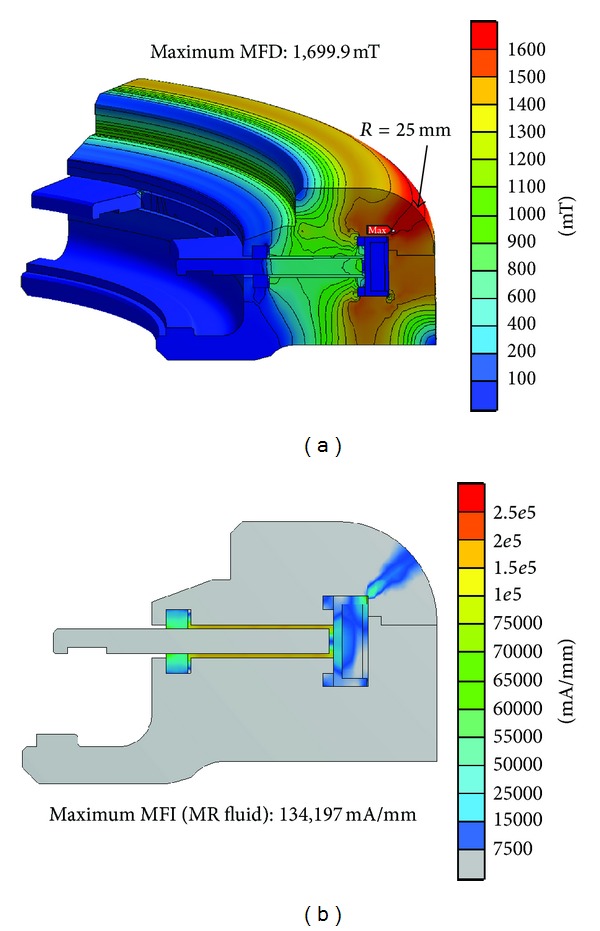
FEA results of the rotary MR damper (Model B, *R* = 25 mm). (a) Magnetic flux density (MFD). (b) Magnetic field intensity (MFI).

A magnetostatic FE analysis of the initial rotary MR damper design (Model A), as described above, resulted in a maximum MFD value of 1.66 T near the coil case of the upper cover case. The sealing area of the flange also showed a high MFD value of 1.62 T (Figure 6(a)). At an applied current of 1.5 A, these MFD values are very close to the saturation limit of SS-400 material (1.6 T), which can cause unstable magnetic behavior of the material. Therefore, the MR damper design needs to be changed to avoid the saturation limit by increasing the cross-sectional area of the ferromagnetic material and thereby deintensifying the MFD. The maximum MFI value was 132,805 mA/mm and occurred in the MR fluid (Figure 6(b)). In this study, the MFI can be used to compute the yield stress of the MR fluid, thus producing the MR damper damping torque capacity. To evaluate damping torque capacity, however, both MFI values of the upper and lower contact areas between the MR fluid and disk should be used, because the MFI value of the upper side is different from that of the lower side due to the asymmetrical shape of the MR damper. In the initial design, the saturated MFD value at the sealing area was overcome by locating the disk and MR fluid positions in the middle of the coil (Figure 7) and by increasing the gap between the MR fluid and the sealing point (panel A in Figure 6(a)). As a result of these changes in Model A, Model B was created.

**Figure 8:**
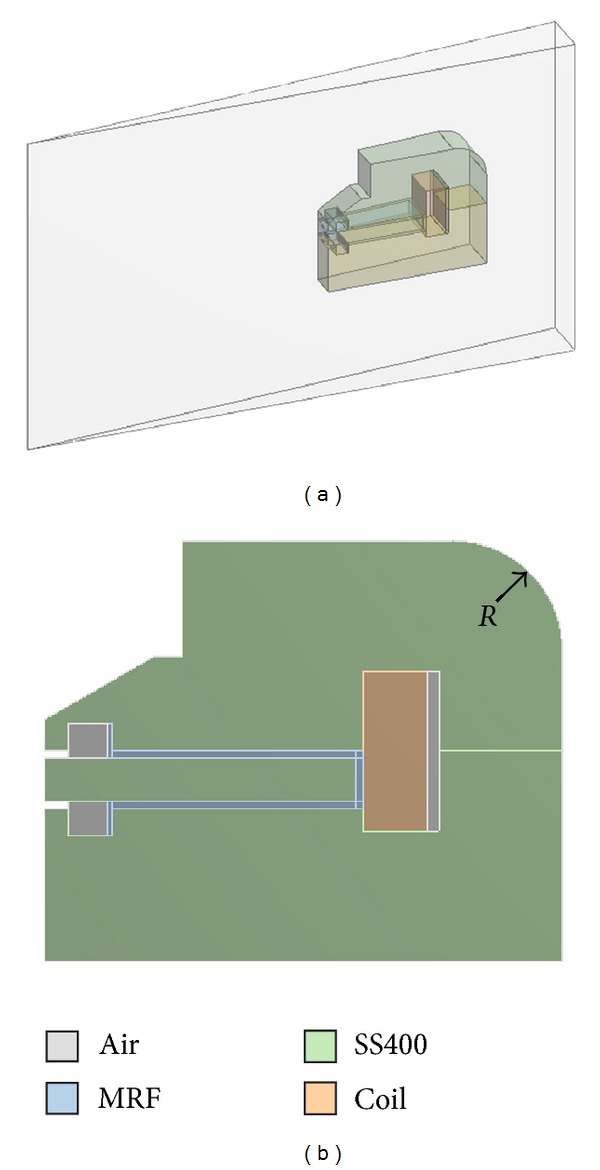
3D model of the rotary MR damper for FE analysis with the curvature, *R* = 5~25 mm. (a) Simplified MR damper model (1/72 symmetric model). (b) Cross-section and component materials.

**Figure 9:**
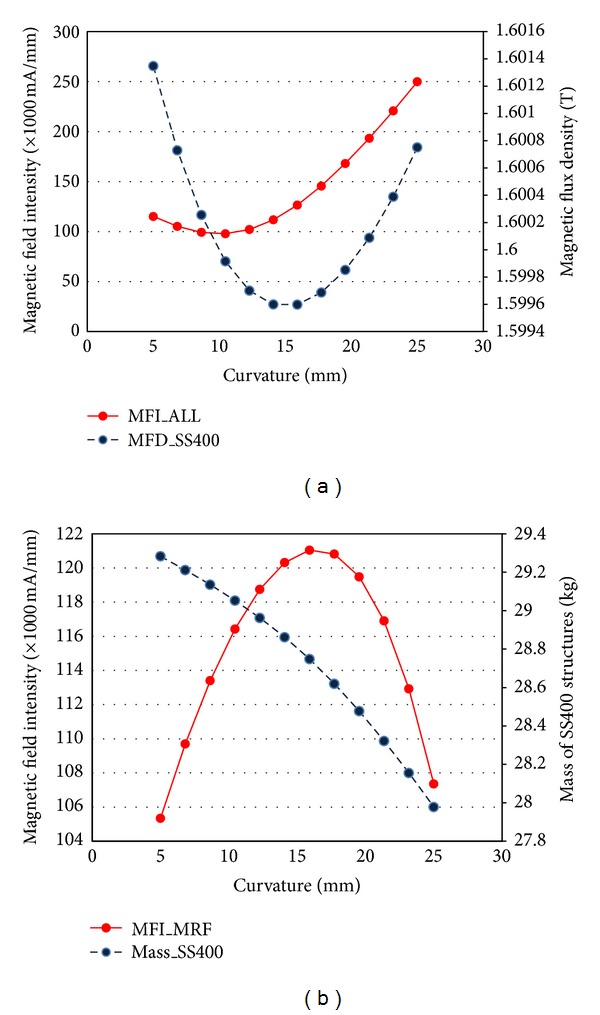
FEA results of the rotary MR damper with the curvature, *R* = 5~25 mm. (a) Maximum magnetic field intensity (MFI) of all components and magnetic flux density (MFD) of the SS400 cover case structure. (b) Average magnetic field intensity (MFI) of the MR fluid (MRF) and mass of the SS400 cover case structure.

Although Model B overcame the sealing area saturation problem of the MFD, the MFD value near the outer surface of the MR damper cover case (~1.7 T) was still saturated, as shown in Figure 7. Therefore, the cross-sectional area of this region further changed within the design limitations of the inner surface curvature of the cover case and the heights of the cover and flange; the design limitations were caused by the dimensions of a rotary MR damper installation space. To change the cross-sectional area, the curvature (*R*) of the upper cover case was selected to be a design parameter, and the corresponding 3D model was constructed (Figure 8). The generated 3D model was cut as 1/72 of the whole model from Model A and simplified by excluding some geometries on disks, sealing parts, and flanges to reduce the calculation time of FE analysis. The parameter *R* value was changed between 5 mm and 25 mm. When the curvature *R* is approximately 5 mm, the MFD value of the cover case structure was lowest by overcoming the saturation limit, 1.6 T (Figure 9(a)). Moreover, the average MFI on the MR fluid, which was extracted from the surface contacted with disks, was highest around *R* = 16 mm (Figure 9(b)). However, the MFI of all components increased with increasing curvature values, while the mass of the cover case decreased with increasing curvatures as expected (Figure 9). Based on these results, a value of *R* = 15 mm is determined for the optimal design of the damper cover case curvature, which could give the best performance for the MFD on the cover case and the MFI on the MR fluid as well as a tradeoff between the MFI of all components and the mass of the cover case. Therefore, the outer surface radius of the cover with the saturated MFD was reduced from 25 mm (Model A and Model B) to 15 mm (Model C) in order to increase the cross-section of the saturated region and thereby decrease the MFD. 

**Figure 10:**
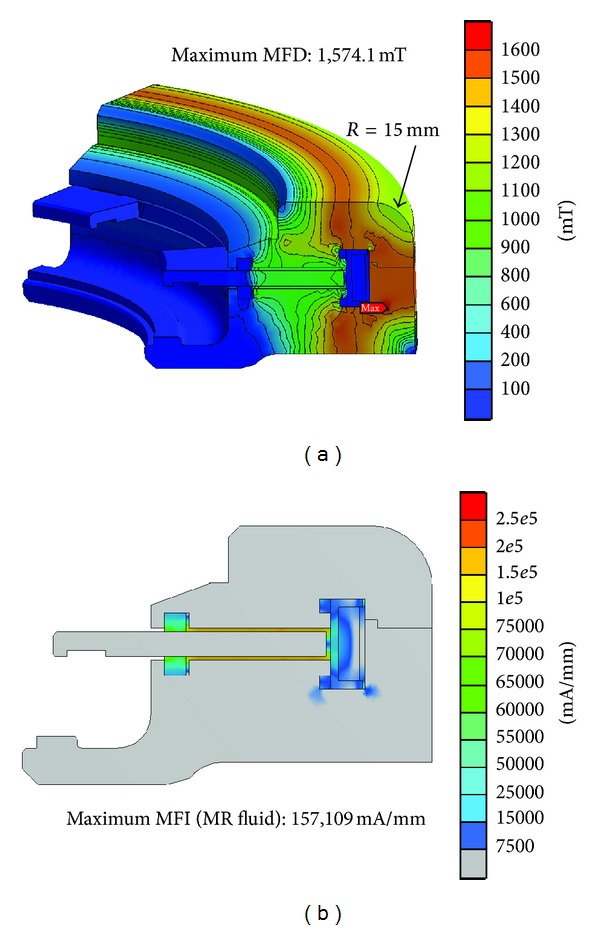
FEA results of the rotary MR damper (Model C, *R* = 15 mm). (a) Magnetic flux density (MFD). (b) Magnetic field intensity (MFI).

The magnetostatic FE analysis of Model C, obtained using the same electric current and boundary conditions as Model A and B, exhibited a maximum MFD value of 1.57 T in the middle of the cover and flange (Figure 10(a)). This value was lower than the saturation limit of the SS-400 material (~1.6 T). The maximum MFI of Model C, which is higher than that of Model B, was 157,109 mA/mm. The higher MFI value resulted in a higher damping torque capacity, as shown by (4). The first objective of this study is that the maximum MFD should be lower than the saturation limits of the component materials, and this objective was achieved using Model C. Therefore, we analyzed Model C to determine if the damping torque capacity would be suitable for unmanned vehicle suspension systems, which is the second objective of our study.

### 4.2. Damping Torque Evaluation

**Figure 11:**
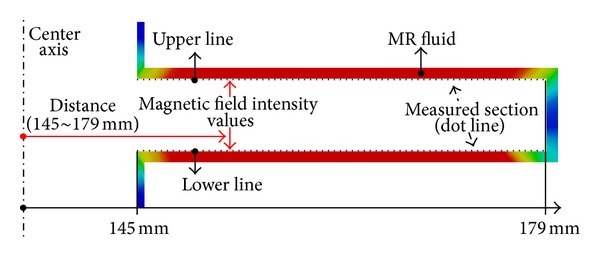
Magnetic field intensity (MFI) extracted from the upper and lower contact lines between the MR fluid and the disk.

**Figure 12:**
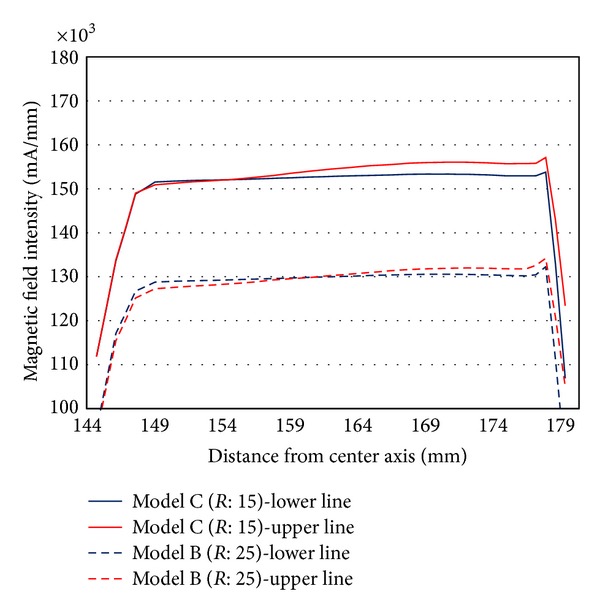
Magnetic field intensity (MFI) as a function of the radial distance from the center axis.

The damping torque capacity of the rotary MR damper was determined from the MFI magnitude on the contact plane between the MR fluid and the disk (this also directly affects the shear stress on the disk's surface). To calculate the damping torque of the MR damper, the MFI values of the upper and lower contact lines between the MR fluid and the disk were extracted from Models B and C (Figure 11) and plotted as a function of the radial distance from the center axis (Figure 12). By changing the outer radius of the cover from *R* = 25 mm (Model B) to *R* = 15 mm (Model C), the average MFI value increased by 22,800 mA/mm. Thus, the damping torque of Model C was expected to be higher than that of Model B.

The MFI values were different between the upper and lower lines although at the same curvature size of the damper cover. Because previous investigators have generally designed MR dampers with symmetrical shapes between the upper and lower sides, both extracted MFI values at the upper and lower lines are equal to each other, and thus either one of the two MFI values calculated at the upper and lower lines can be used. In this study, however, the asymmetry in the MR damper against the horizontal plane led to two different MFI values between the upper and lower lines, and the use of both different MFI values enhanced the accuracy of the damping torque evaluation in the present study. There was a tendency for the MFI of the upper to be higher than that of the lower with decreasing damper curvature size as well as both MFI values of the upper and lower to increase with increasing distances from the center axis.

The damping torques of the rotary MR damper calculated using both MFI values of the upper and lower contact region (Figure 12) and the characteristic equation of the MR fluid (1) were 239.2, 436.95, and 576.78 N·m at currents of 0.5, 1, and 1.5 A, respectively, for a disk rotating speed of 10 RPM. The damping torque satisfied the required value of damping torque of 475 N·m at 1.5 A at a rotating speed of 10 RPM, and its magnitude increased with increasing current, although the increment was gradually reduced with increasing current.

## 5. Experimental Validation of the Proposed Rotary MR Damper

**Figure 13:**
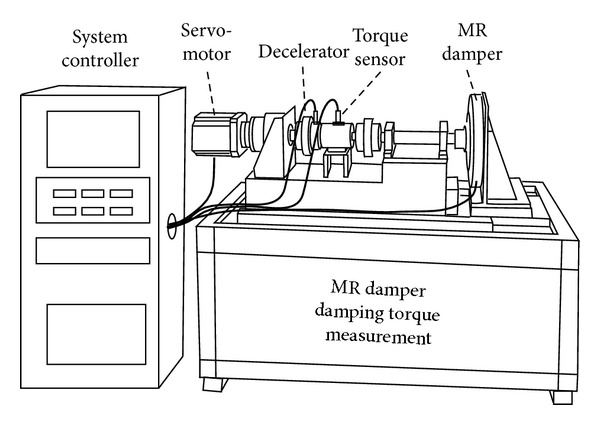
Apparatus for performing damping torque measurement tests.

**Figure 14:**
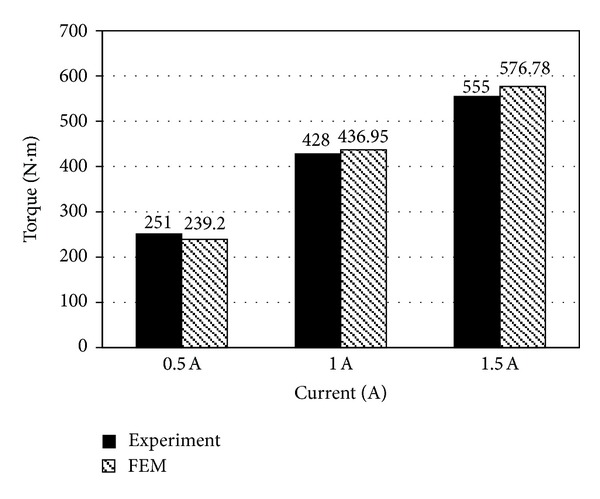
Comparison of the analytical and experimental damping torque values.

The damping torque was experimentally measured by changing the current using the custom-built testing apparatus shown in Figure 13. This apparatus consisted of a servo-motor (HC-RFS153, Mitsubishi, Japan) and a decelerator (PGX142, Dae-sung PTS, Korea) connected in series with a torque sensor (T22, Hottinger Baldwin Messtechnik GmbH, Germany). To measure the damping torque of the rotary MR damper, the servo-motor was controlled at a constant speed of 10 RPM using a motor driver (MR-J2S-200A, Mitsubishi, Japan) and a Programmable Logic Controller (PLC) (FX3u-16 M, Mitsubishi, Japan). A torque sensor installed between the servo-motor and the rotary MR damper was used to measure the resisting torque of the servo-motor to overcome a reduction in motor speed, due to an increase in the viscosity of the MR damper with current supplied by an MR damper controller (TMS320LF2406 DSP, Texas Instruments, USA). The experimentally measured damping torques were 251, 428, and 555 N·m at currents of 0.5, 1, and 1.5 A, respectively, and the values were comparable with the analytically predicted values with errors of 4.70, −2.09, and −3.92% at 0.5, 1, and 1.5 A, respectively (Figure 14).

## 6. Conclusions

Using magnetostatic 3D FE analysis and the MR fluid characteristic equation, we successfully designed a rotary MR damper with a specified damping torque capacity and unsaturated magnetic flux density (MFD) for unmanned vehicle suspension systems. The present study revealed the effect of the sealing location and the cover case curvature on the performance of the rotary type MR damper and optimized the curvature of the MR damper cover case. The small cross-sectional area of the sealing region in the flange, due to the narrow gap between the rotation disk and the sealing ring, resulted in the saturated MFD. This saturated sealing region MFD was reduced below the saturation limit of the material by locating the disk and MR fluid positions in the middle of the coil and by increasing the gap between the MR fluid and the sealing point. Moreover, the MFD was very sensitive to the curvature of the MR damper cover case, and the saturated MFD in the radial region between the coil case and the MR damper cover could be addressed by adjusting the curvature of the damper cover case. This curvature was further optimized by considering the MFD, the magnetic field intensity (MFI), and the weight of the MR damper components.

The damping torque of the rotary MR damper was predicted using the MFI of the MR damper and the characteristic equation of the MR fluid, with errors of less than 5% when compared to experimental measurements from the rotary MR damper, which was manufactured by our proposed design, using a custom-made testing apparatus. Here, the accuracy of the analytically predicted damping torque was improved due to the use of both MFI values of the asymmetric upper and lower contact areas between the MR fluid and disk as well as the use of the much detailed 3D MR damper model on FE analysis. The results of the present study could play an important role in the design of rotary MR dampers by reducing product development time and cost while maintaining a high level of performance.
